# Endotoxic shock-expanded murine CD11c^low^CD45RB^+^ regulatory dendritic cells modulate inflammatory T cell responses through multiple mechanisms

**DOI:** 10.1038/srep10653

**Published:** 2015-05-29

**Authors:** Xiaoqian Wang, Qingyang Wang, Xueying Zhang, Yajing Li, Jingyang Wang, Chunmei Hou, Juan Chen, Beifen Shen, Yanchun Shi, Jiyan Zhang

**Affiliations:** 1 Department of Molecular Immunology, Institute of Basic Medical Sciences, 27 Taiping Road, Beijing 100850, P. R. China; 2 Research Center of Molecular Biology, Inner Mongolia Medical College, Hohhot 010059, P. R. China

## Abstract

Changes in the number and function of dendritic cells (DCs) have been reported to play an important role in endotoxin tolerance. It has been reported that expansion of splenic CD11c^low^CD45RB^+^ DCs occurs in mice injected with sublethal doses of lipopolysaccharide (LPS). However, the function of endotoxic shock-expanded CD11c^low^CD45RB^+^ DCs has not been examined. In this work, we show that endotoxic shock promotes the expansion of CD11c^low^CD45RB^+^ cells with dendritic morphology and the production of low levels of inflammatory cytokines and costimulatory molecules. The expanded cells induce the generation of regulatory T cells (Tregs), show incapability to stimulate T cells, and induce apoptosis of CD4^+^ T cells *in vitro*. As compared to CD11c^hi^CD45RB^−^ conventional DCs, the expanded cells exert better protection against colitis induction by CD4^+^ CD25^−^ T cells, even though both subpopulations show similar ability to induce Tregs *in vivo.* The better control of proinflammatory cytokine responses *in vivo* by the expanded cells is associated with more apoptosis in the Payer’s patches and in colonic tissue-infiltrating cells. Thus, the expanded cells can modulate inflammatory T cell responses through multiple mechanisms. Our study facilitates a better understanding how innate immune responses may shape adaptive immunity and immune suppression following LPS-induced acute inflammation.

Early recognition of invading bacteria by the innate immune system fundamentally contributes to antibacterial defence by triggering inflammatory responses that prevent the spread of infection and suppress bacterial growth^1^,^2^,^3^. A key step in the induction of the inflammatory response to gram-negative bacteria is the activation of Toll-like receptor 4 (TLR4) signalling by lipopolysaccharide (LPS), a major component of the outer membrane of all gram-negative bacteria^1^,^2^,^3^. Dendritic cells (DCs), defined by their dendritic morphology and unique phenotype, are involved in the initiation of inflammation in response to gram-negative bacteria^3^,^4^,^5^. Moreover, DCs are the most potent professional antigen-presenting cells and thus play a pivotal role in linking the innate and adaptive immune response^3^,^4^,^5^. In addition to their vital roles in antibacterial defence, DCs are also indispensable for the induction and maintenance of immunological tolerance. Recently, the identification and characterisation of DCs with regulatory properties (so-called regulatory or tolerogenic DCs) has attracted much attention^4^,^5^,^6^,^7^,^8^,^9^,^10^. Regulatory DCs usually produce large amounts of interleukin-10 (IL-10), thereby promoting the generation of IL-10-producing T cells^6^,^7^,^8^,^9^,^10^. However, whether regulatory DCs can modulate inflammatory T cell responses through other mechanisms remains unclear.

Several reports have discussed the potential regulatory function of a DC subset characterised by its particular CD11c^low^CD45RB^+^ surface marker expression^6^,^7^,^8^,^9^,^10^. Naturally occurring CD11c^low^CD45RB^+^ DCs are present in the spleens and lymph nodes of normal mice and are present at an increased level in transgenic mice expressing high levels of IL-10 and in mice experiencing a parasitic infection^6^,^10^. Naturally occurring CD11c^low^CD45RB^+^ DCs and those induced by a parasitic infection have been demonstrated to induce IL-10-expressing CD4^+^ T cells^6^,^10^. A similar expansion of splenic CD11c^low^CD45RB^+^ DCs has also been reported in mice injected with sublethal doses of LPS^10^. Changes in the number and function of DCs have been reported to play an important role in endotoxin tolerance^4^,^5^. However, the function of endotoxic shock-expanded CD11c^low^CD45RB^+^ DCs has not been examined.

In this work, we show that intra-peritoneal (i.p.) *Escherichia coli* (*E. coli*) infection and i.p. administration of *E. coli*-derived LPS both induce the expansion of CD11c^low^CD45RB^+^ cells with dendritic morphology and the production of low levels of inflammatory cytokines and costimulatory molecules. The expanded CD11c^low^CD45RB^+^ DCs modulate inflammatory T cell responses through induction of regulatory T cells (Tregs), incapability to stimulate T cells, and induction of T cell apoptosis.

## Results

### Endotoxic shock promotes the expansion of CD11c^low^CD45RB^+^ cells

**Figure 1:**
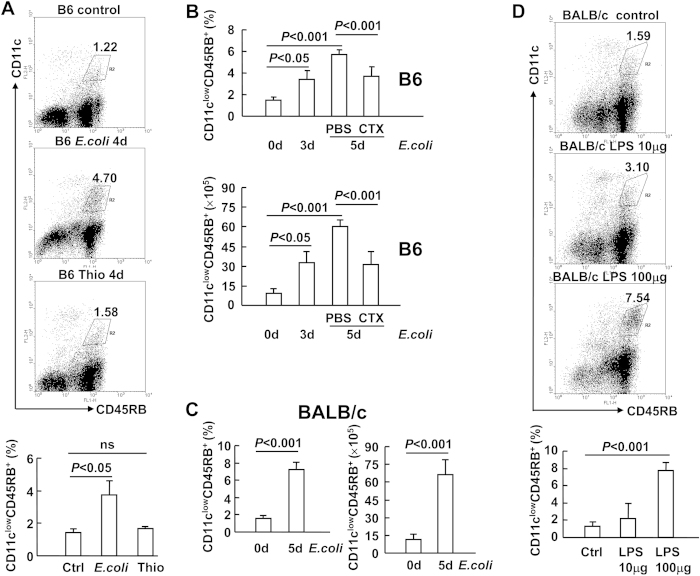
Endotoxic shock promotes the expansion of CD11c^low^CD45RB^+^ cells. *A*, B6 mice were intra-peritoneally (i.p.) injected with *Escherichia coli* (*E. coli*) K12 or thioglycolate. Four days later, mice were euthanised and the splenic cells were subjected to flow cytometry analysis of CD11c and CD45RB expression. ns, not significant. *B*, B6 mice were i.p. injected with cholera toxin CHX, (5 μg/mouse) or PBS of equal volume, followed by i.p. injection of *E. coli* K12 one hour later. Mice were euthanised at the indicated time points, and the splenic cells were subjected to flow cytometry analysis of CD11c and CD45RB expression. *C* and *D*, BALB/c mice were i.p. injected with *E. coli* K12 or *E. coli*-derived LPS. The splenic cells were subjected to flow cytometry analysis of CD11c and CD45RB expression 5 days later.

With antibodies against CD11c and CD45RB, we identified three subpopulations of CD11c-expressing cells, CD11c^hi^CD45RB^−^, CD11c^low^CD45RB^+^ , and CD11c^low^CD45RB^−^, in the spleens of C57BL/6 (B6) mice (Fig. 1A). To verify whether endotoxic shock leads to the expansion of CD11c^low^CD45RB^+^ cells, we used the laboratory *E. coli* strain K12. Four days after i.p. infection with *E. coli* K12, the percentage of CD11c^low^CD45RB^+^ cells, but not of the other subpopulations, increased (Fig. 1A). However, in a model of acute self-limiting sterile inflammation^11^, the percentage of CD11c^low^CD45RB^+^ cells remained largely unchanged 4 days after i.p. injection of thioglycolate (Fig. 1A). These data suggest that the expansion of CD11c^low^CD45RB^+^ cells depends on the intensity of inflammation. Because of the splenomegaly induced by *E. coli* infection, the absolute number of CD11c^low^CD45RB^+^ cells increased over 5-fold, reaching its peak on day 5 after infection (Fig. 1B). This expansion was significantly reduced by simultaneous treatment with cholera toxin (Fig. 1B), which has been shown to suppress inflammation *in vivo*^12^, further suggesting that the intensity of inflammation is indispensable for the expansion of CD11c^low^CD45RB^+^ cells. The expansion of these cells also occurred in BALB/c mice (Fig. 1C). A similar change in splenic DC subsets was observed when mice were injected with sublethal doses of purified *E. coli* LPS (Fig. 1D). Therefore, endotoxic shock promotes the expansion of CD11c^low^CD45RB^+^ cells.

### Phenotypic characterisation of the expanded CD11c^low^CD45RB^+^ cells

**Figure 2:**
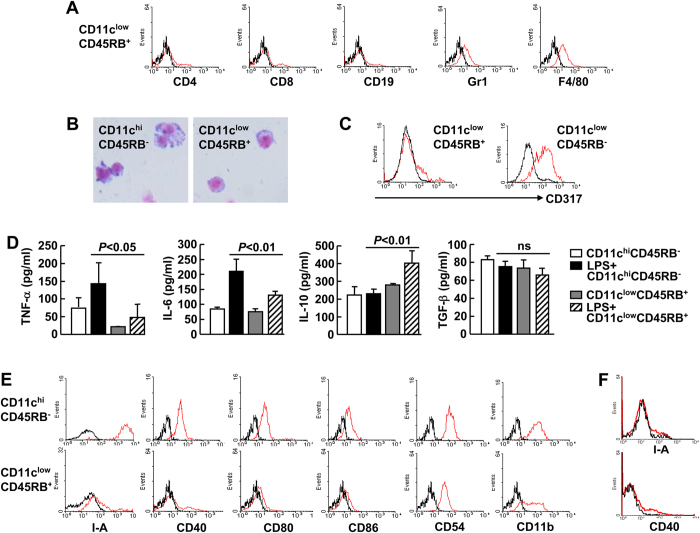
Phenotypic characterisation of the expanded CD11c^low^CD45RB^+^ cells. BALB/c mice were intra-peritoneally (i.p.) injected with *E. coli*-derived LPS. Five days later, mice were euthanised and the splenic cells were subjected to the following analyses. ***A***, ***C***, and ***E***, The expression of lineage markers and functional molecules on the surface of different subpopulations of CD11c^+^ cells was analysed by flow cytometry. The black lines represent cells stained with isotype antibodies. *B*, *D*, and *F*, CD11c^low^CD45RB^+^ cells or CD11c^hi^CD45RB^−^ cells were purified, the morphology was analysed by Giemsa staining (***B***), cytokine profiles of the two subpopulations stimulated with or without 0.5 μg/ml LPS for 24 h were analysed by ELISA (***F***), and the expression of functional molecules on the surface of CD11c^low^CD45RB^+^ cells stimulated with 0.5 μg/ml LPS for 24 h was analysed by flow cytometry. The black lines represent cells stained with isotype antibodies (***F***).

Next, we attempted to identify the expanded CD11c^low^CD45RB^+^ cells. Regarding lineage markers, these cells showed weak expression of Gr1 and F4/80 and no expression of the T cell markers CD4 and CD8 or the B cell marker CD19 (Fig. 2A). Giemsa staining revealed the expanded cells exhibited immature dendritic morphology, as compared to the mature dendritic morphology of CD11c^hi^CD45RB^−^ conventional DCs (Fig. 2B). As plasmacytoid DCs also express low levels of CD11c, we stained splenocytes from mice using the plasmacytoid DC marker CD317 (also called PDCA-1)^13^. The expanded cells did not express CD317, but CD11c^low^CD45RB^−^ cells exhibited significant expression of that marker (Fig. 2C). The analysis of the cytokine profiles revealed that the expanded cells secreted slightly less tumor necrosis factor-α (TNF-α) and IL-6, a similar level of transforming growth factor-β (TGF-β), and a slightly higher level of IL-10 in response to LPS as compared to CD11c^hi^CD45RB^−^ conventional DCs (Fig. 2D). Regarding functional markers, the expanded cells showed almost undetectable levels of major histocompatibility complex (MHC) molecule I-A and costimulatory molecules (CD40, CD80, CD86), in contrast to the significant expression of these molecules in CD11c^hi^CD45RB^−^ conventional DCs (Fig. 2E). In addition, the expanded cells expressed the adherence molecules CD54 and CD11b, but to a lesser extent than CD11c^hi^CD45RB^−^ conventional DCs (Fig. 2E). MHC molecule I-A and costimulatory molecule CD40 remained undetectable after the expanded cells underwent *in vitro* stimulation with LPS, indicating a stable phenotype for these cells (Fig. 2F). Taken together, these data suggest that endotoxic shock-expanded CD11c^low^CD45RB^+^ cells are less capable of stimulating T cells than CD11c^hi^CD45RB^−^ conventional DCs. On the other hand, only 15% of CD11c^low^CD45RB^+^ cells in untreated mice showed low level of MHC molecule I-A expression (Supplementary Figure 1). Moreover, the majority of CD11c^low^CD45RB^+^ I-A^−^ cells purified from untreated mice upregulated the expression of MHC molecule I-A after they underwent *in vitro* stimulation with LPS (Supplementary Figure 2). These data suggest naturally occurring CD11c^low^CD45RB^+^ cells are heterogeneous and only a small portion of them have regulatory effects. Therefore, it is more interesting to explore the functions of the expanded CD11c^low^CD45RB^+^ cells.

### The expanded CD11c^low^CD45RB^+^ cells induce apoptosis of CD4^+^ T cells *in vitro*

**Figure 3:**
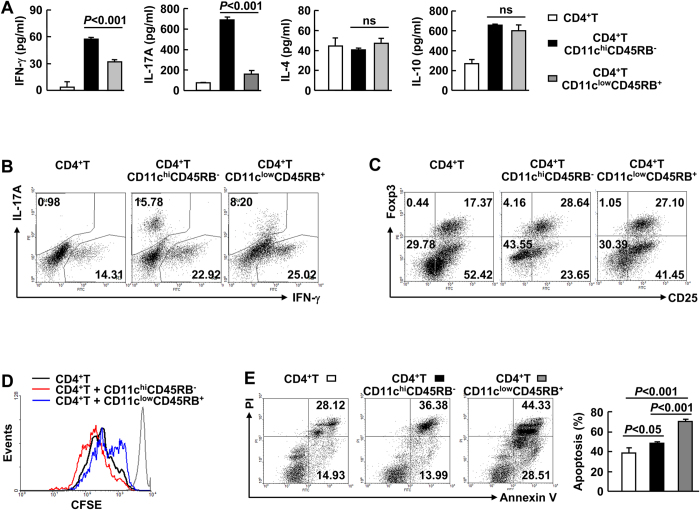
The expanded CD11c^low^CD45RB^+^ cells induce apoptosis of activated CD4^+^ T cells *in vitro*. Purified CD11c^low^CD45RB^+^ cells or CD11c^hi^CD45RB^−^ cells were co-cultured with CD4^+^ CD25^−^ splenic cells at the ratio of 4:1. Stimulation was affected by the Dynabeads mouse CD3/CD28 T cell expander. *A*-*C*, The co-culture was subjected to Th0 (***A***), Th1 (***B***), or Tregs (***C***) condition. Five days later, the supernatants were subjected to ELISA (***A***). IFN-γ, IL-17, Foxp3, or CD25 expression in CD4^+^ cells was analysed by flow cytometry (***B*** and ***C***). *D*, CD4^+^ CD25^−^ splenic cells were labelled with CFSE before co-culture. Proliferation was assessed by flow-cytometric analysis of CFSE dilution 96 hours later. The grey line represents cells with CFSE labelling but without proliferation. *E*, Apoptosis was assessed by flow-cytometric analysis of the percentages of CD4^+^ Annexin-V^+^ cells 96 hours later. *Left*, representative data; *right*, statistical data (n = 3).

Because we did not observe an inhibitory role of the expanded CD11c^low^CD45RB^+^ cells on cytokine production by macrophages *in vitro* (data not shown), we next explored how the expanded cells might affect T cell responsiveness. The potential mechanisms of inflammatory T cell responses include molecular mimicry, bystander activation, epitope spreading, and superantigen activation of T cells^14^,^15^,^16^. In this regard, a polyclonal T cell stimulation approach was employed. Allogeneic CD4^+^ CD25^−^ T cells were co-cultured with the expanded CD11c^low^CD45RB^+^ cells or CD11c^hi^CD45RB^−^ conventional DCs. Stimulation was performed using beads conjugated with antibodies against CD3 and CD28 in the presence of neutralisation antibodies against IFN-γ and IL-4. After 5 days, secretion of IFN-γ, IL-4, IL-10, and IL-17A was evaluated by ELISA (Fig. 3A). Co-culture of allogeneic CD4^+^ CD25^−^ T cells with conventional DCs significantly enhanced IFN-γ and IL-17A secretion and slightly enhanced IL-10 secretion without affecting IL-4 production (Fig. 3A). Co-culture of allogeneic CD4^+^ CD25^−^ T cells with the expanded cells led to a partial decrease in IFN-γ secretion, similar IL-4 and IL-10 production, and a dramatic decrease in IL-17A secretion compared to co-culture with conventional DCs (Fig. 3A). Intracellular staining revealed that under the Th1 condition, co-culture with conventional DCs significantly augmented the proportion of CD4^+^ T cells that produced IFN-γ or IL-17A (Fig. 3B). Co-culture of allogeneic CD4^+^ CD25^−^ T cells with the expanded cells resulted in a decreased proportion of CD4^+^ T cells producing IL-17A but a similar proportion of CD4^+^ T cells producing IFN-γ as compared to co-culture with conventional DCs (Fig. 3B). In addition, the expanded cells showed similar ability to promote Foxp3 expression compared with conventional DCs under both Th0 condition (Supplementary Figure 3) and the Treg condition (Fig. 3C).

The similar percentages of CD4^+^ T cells expressing IFN-γ and the different IFN-γ levels in the supernatants obtained from the two co-culture systems suggest that the expanded cells might affect T cell proliferation and/or survival. *In vitro* CFSE dilution analysis revealed that the antigen-nonspecific proliferation of CD4^+^ T cells was slightly enhanced by conventional DCs and slightly inhibited by the expanded cells (Fig. 3D). Apoptosis analysis demonstrated that conventional DCs slightly promoted apoptosis of CD4^+^ T cells, but the expanded cells exhibited a much stronger ability to promote apoptosis (Fig. 3E; Supplementary Figure 4). Taken together, these data suggest that the expanded cells partially affect T cell responsiveness *in vitro*.

### Fas ligand (FasL) mediates the induction of CD4^+^ T cell apoptosis by expanded CD11c^low^CD45RB^+^ cells *in vitro*

**Figure 4:**
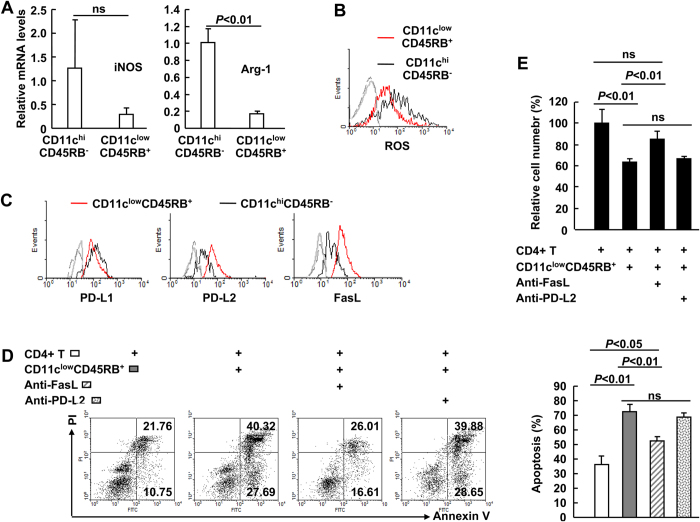
Fas ligand (FasL) mediates the expanded CD11c^low^CD45RB^+^ cell-induced apoptosis of CD4^+^ T cell *in vitro*. ***A*** Purified CD11c^low^CD45RB^+^ cells and CD11c^hi^CD45RB^−^ cells were subjected to real-time PCR. *B* and *C*, The levels of ROS (***B***) and cell surface markers (***C***) in different subpopulations of CD11c^+^ cells were analysed by flow cytometry. The grey lines represent background fluorescence (***B***) or staining with isotype antibodies (***C***). *D* and *E*, Purified CD11c^low^CD45RB^+^ cells and CD11c^hi^CD45RB^−^ cells were co-cultured with CD4^+^ CD25^−^ splenic cells at the ratio of 4:1. Stimulation was affected by Dynabeads mouse CD3/CD28 T cell expander in the presence or absence of neutralisation antibodies against programmed death ligand 2 (PD-L2) and FasL. Ninety-six hours later, cells were stained with anti-CD4-PECy5, PI, and Annexin V-FITC resuspended in 300 μl binding buffer containing calcium ion. Apoptosis was assessed by flow-cytometric analysis of the percentages of CD4^+^ Annexin-V^+^ cells. *Left*, representative data; *right*, statistical data (n = 3) (***D***). Cells were collected using a FACSCalibur flow cytometer at high speed for 60 seconds, and the number of CD4^+^ Annexin-V^−^ cells was counted. The results are expressed as the percentage of basal growth of CD4^+^ T cells (***E***).

Because the expanded CD11c^low^CD45RB^+^ cells did not show significantly higher production of IL-10 or TGF-β as compared to CD11c^hi^CD45RB^−^ conventional DCs (Fig. 2D), these expanded cells must employ other mechanism(s) to affect T cell responsiveness *in vitro*. Multiple molecules, including nitric oxide, reactive oxygen species (ROS), programmed death ligand 1 (PD-L1), PD-L2, and Fas ligand (FasL), have been reported to trigger apoptosis of activated CD4^+^ T cells^17^,^18^,^19^,^20^. The expanded CD11c^low^CD45RB^+^ cells did not exhibit higher levels of inducible nitric oxide synthase (iNOS), ROS, or PD-L1 than CD11c^hi^CD45RB^−^ conventional DCs (Fig. 4A–C). Consistent with the proliferation data, the expanded cells also did not show enhanced production of arginase I (Fig. 4A), which has been reported to mediate the inhibition of T cell proliferation^16^. However, the expanded cells expressed higher levels of PD-L2 and FasL than conventional DCs (Fig. 4C). With neutralisation antibodies against PD-L2 and FasL, we tested the roles of these two molecules in the induction of apoptosis. As expected, the blockade of FasL significantly reversed the enhanced apoptosis (Fig. 4D) and the reduced cell number (Fig. 4E) of CD4^+^ T cells co-cultured with the expanded cells, whereas the blockade of PD-L2 exhibited only a marginal effect (Fig. 4D,E). Thus, FasL mediates the induction of CD4^+^ T cell apoptosis by expanded CD11c^low^CD45RB^+^ cells *in vitro*.

### Expanded CD11c^low^CD45RB^+^ DCs suppress colitis induction by CD4^+^ CD25^−^ T cells *in vivo*

**Figure 5:**
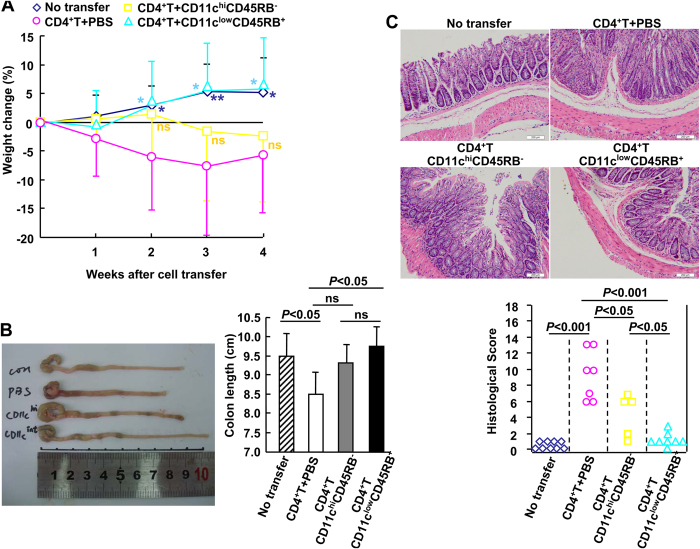
The expanded CD11c^low^CD45RB^+^ dendritic cells (DCs) suppress colitis induction by CD4^+^ CD25^−^ T cells *in vivo*. *A*, Relative changes in percent body weight over time for no-transfer mice (◊, n = 9) and for colitis-induced mice (transfer of CD4^+^ CD25^−^ T cells) subsequently treated with PBS (o, n = 7), CD11c^hi^CD45RB^−^ cells (□, n = 5), or CD11c^low^CD45RB^+^ cells (∆, n = 8). *B*, Left, macroscopic findings of the colon on day 28 after transfer of CD4^+^ CD25^−^ T cells. Right, the colon lengths of colitic mice were measured on day 28. *C*, Top, histological analysis of the colon was carried out on day 28. Scale bar: 50 μM. Bottom, histological scores of the colons from CD4^+^ CD25^−^ T cell-transferred mice. The results were interpreted independently by two pathologists who were not given previous information regarding the treatment of the mice. Two independent experiments gave similar results.

To verify whether endotoxic shock-expanded CD11c^low^CD45RB^+^ cells partially suppress T cell responsiveness *in vivo*, colitis induction by CD4^+^ CD25^−^ T cells was employed^21^,^22^. CB-17 SCID mice were divided into four groups, and CD4^+^ CD25^−^ colitogenic effector T cells were transferred into three of the groups to induce colitis, as described previously^21^,^22^. At the time of T cell transfer, mice were treated with PBS containing CD11c^hi^CD45RB^−^ conventional DCs or the expanded CD11c^low^CD45RB^+^ DCs (1 × 10^6^ cells/mouse). Four weeks after T cell transfer, mice treated with CD4^+^ CD25^−^ T cells/PBS showed significantly lower body weights than untreated mice (Fig. 5A). Treatment with CD11c^hi^CD45RB^−^ conventional DCs was insufficient for protection against the loss of body weight (Fig. 5A). However, treatment with the expanded CD11c^low^CD45RB^+^ DCs significantly reversed the loss of body weight (Fig. 5A). Macroscopic examinations at week 4 after transfer revealed that treatment with CD11c^hi^CD45RB^−^ conventional DCs tended to reverse the shortening of the colon but not at a statistically significant level (Fig. 5B). In contrast, treatment with the expanded cells led to significantly longer colons (Fig. 5B). Upon histological examination, colitis was characterised by severe epithelial hyper-proliferation, mucus depletion, massive infiltration of inflammatory cells, crypt abscesses, reduced numbers of goblet cells, and erosions. CB-17 SCID mice injected with CD4^+^ CD25^−^ T cells and treated with PBS had severe colitis (Fig. 5C). Treatment with CD11c^hi^CD45RB^−^ conventional DCs partially improved these histological signs (Fig. 5C). More importantly, treatment with the expanded cells almost completely abolished signs of colitis (Fig. 5C). There was a significant reduction of inflammatory cell infiltration, and goblet cell number and mucus were preserved at normal levels (Fig. 5C). To rule out the possibility that the DC preparations contained residual T cells that were responsible for the protective effects in the co-transfer experiment, rather than the DCs, SCID mice were used as recipients of the expanded cells. Four weeks later, flow cytometry revealed that no CD4+ or CD3+ cells could be detected in the spleens of SCID mice with or without transfer of the expanded cells (Supplementary Figure 5). Thus, the expanded CD11c^low^CD45RB^+^ DCs suppressed colitis induction by CD4^+^ CD25^−^ T cells *in vivo*.

### Administration of expanded CD11c^low^CD45RB^+^ DCs reduces proinflammatory cytokine responses *in vivo*

**Figure 6:**
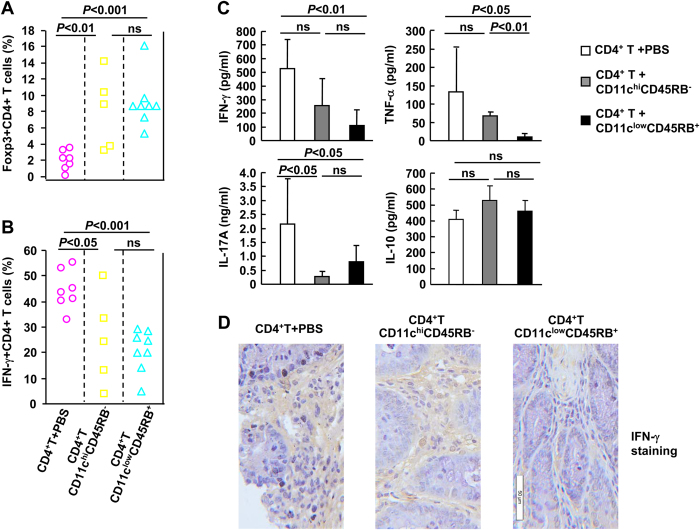
Administration of the expanded CD11c^low^CD45RB^+^ dendritic cells (DCs) reduces proinflammatory cytokine responses *in vivo*. Mice were treated as described in Fig. 5A. The percentages of Foxp3^+^ CD4^+^ (***A***) or IFN-γ^+^ CD4^+^ (***B***) splenic T cells were analysed by flow cytometry on day 28. Mesenteric lymph node (MLN) cells (1 × 10^6^) of colitic mice were cultured for 24 h in 24-well plates in the presence of 1 μg/ml CD3 and 1 μg/ml CD28. The culture supernatants were then harvested and subjected to ELISA (***C***). The colon samples of colitic mice were subjected to immunohistochemical staining with a primary antibody against IFN-γ. Scale bar: 50 μM (***D***).

To elucidate the mechanism(s) underlying the protective role of the expanded cells against colitis, the expression levels of Foxp3 and proinflammatory cytokines were measured. Treatment with either CD11c^hi^CD45RB^−^ conventional DCs or the expanded CD11c^low^CD45RB^+^ DCs significantly induced Foxp3 expression in splenic CD4^+^ T cells (Fig. 6A). Furthermore, treatment with either of the two cell types led to reduced Th1 differentiation in spleens, as revealed by intracellular IFN-γ staining (Fig. 6B). However, there was no significant difference in Foxp3 or IFN-γ expression in splenic CD4^+^ T cells between the groups of mice treated with conventional DCs and those treated with the expanded cells (Fig. 6A,B). To get a better idea about the proinflammatory cytokine responses *in vivo*, mesenteric lymph node (MLN) cells (1 × 10^6^) of colitic mice were cultured for 24 h in 24-well plates in the presence of 1 μg/ml CD3 and 1 μg/ml CD28. The culture supernatants were then harvested and subjected to ELISA. As shown in Fig. 6C, treatment with the expanded CD11c^low^CD45RB^+^ DCs, but not treatment with CD11c^hi^CD45RB^−^ conventional DCs, led to significantly reduced IFN-γ and TNF-α production, even though both conditions resulted in significantly reduced IL-17A production. In addition, neither treatment showed an effect on IL-10 expression by MLN cells (Fig. 6C). Since colitis is a colonic inflammatory disease, it would be more meaningful to determine proinflammatory cytokine responses in the colonic tissues. Indeed, immunohistochemistry revealed significantly reduced IFN-γ expression in the colonic tissues upon treatment with the expanded CD11c^low^CD45RB^+^ DCs, but not treatment with CD11c^hi^CD45RB^−^ conventional DCs (Fig. 6D).

### Administration of expanded CD11c^low^CD45RB^+^ DCs induces T cell apoptosis *in vivo*

**Figure 7:**
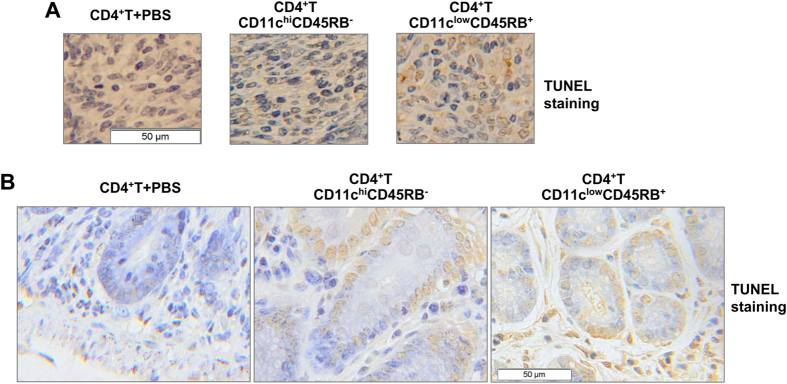
Administration of expanded CD11c^low^CD45RB^+^ dendritic cells (DCs) induces T cell apoptosis *in vivo*. Mice were treated as described in Fig. 5A. The colon samples of colitic mice were subjected to terminal deoxynucleotidyl transferase-mediated dUTP nick end-labelling (TUNEL) staining and the apoptosis in the Payer’s patches (***A***) and the colonic tissues (***B***) was shown. Scale bar: 50 μM.

Since the expanded CD11c^low^CD45RB^+^ cells significantly induce apoptosis of CD4^+^ T cells *in vitro* (Fig. 3E), the better control of proinflammatory cytokine responses by the expanded cells as compared to the conventional DCs might be attributed, at least partially, to their ability to induce T cell apoptosis. To test this notion, the colon samples of colitic mice were subjected to terminal deoxynucleotidyl transferase-mediated dUTP nick end-labelling (TUNEL) staining. As expected, more apoptosis was observed in the Payer’s patches (Fig. 7A) and in colonic tissue-infiltrating cells (Fig. 7B) of mice treated with the expanded cells than in those treated with conventional DCs. Another issue of interest is how long these expanded cells could provide protection. The percentage of this subpopulation in the spleen and MLN was examined until 2 weeks after LPS challenge. Flow cytometry analysis revealed that even though the expanded cells disappear in the mouse spleen after reaching their peak on day 4-5 of LPS treatment (Fig. 1B and Supplementary Figure 6), an elevated percentage of this subpopulation was still detected in mouse MLN cells 10-14 days after LPS treatment (Supplementary Figure 6). Thus, the expanded cells can last for a significant amount of time after endotoxic shock.

## Discussion

Recent work has provided evidence supporting the importance of DCs, especially regulatory DCs, in peripheral tolerance and thus as potential immunotherapies for autoimmune diseases. Regulatory DCs usually produce a large amount of IL-10, thereby promoting the generation of IL-10-producing T cells. However, in this study, endotoxic shock-expanded CD11c^low^CD45RB^+^ regulatory DCs exhibited low levels of basal IL-10 expression and LPS-induced IL-10 expression (Fig. 2D). Consequently, the expanded cells failed to potently induce IL-10 production by T cells *in vitro* (Fig. 3A) and *in vivo* (Fig. 6C). Thus, the expanded cells ameliorated colitis without expressing an abundance of IL-10.

These results led us to examine how these expanded CD11c^low^CD45RB^+^ DCs modulate inflammatory T cell responses, and our results suggest that at least three factors are involved. First, the expanded cells induce the generation of Foxp3+ Tregs. Administration of the expanded cells led to an increase in Tregs *in vivo* (Fig. 6A). *In vitro*, the expanded cells promoted Foxp3 expression under both the Th0 condition (Supplementary Figure 3) and Treg condition (Fig. 3C). Because Tregs play important roles in reducing colitis^23^, it is reasonable to propose that the expanded cells inhibit inflammatory T cell responses through, at least partially, the promotion of the generation of Tregs. CD11c^hi^CD45RB^−^ conventional DCs showed a similar ability to induce Foxp3 expression *in vitro* and *in vivo* (Fig. 3C, Supplementary Figure 3, and Fig. 6A). The induction of Tregs by splenic conventional DCs has been reported to contribute to peripheral tolerance^24^. Consistently, CD11c^hi^CD45RB^−^ conventional DCs exhibited partial protection against the induction of colitis by CD4^+^ CD25^−^ T cells (Fig. 5), even though these conventional DCs significantly augmented IFN-γ and IL-17A expression in activated CD4^+^ T cells (Fig. 3A,B) and slightly enhanced the antigen-nonspecific proliferation of CD4^+^ T cells (Fig. 3D) *in vitro*. *In vivo*, the induction of Tregs by conventional DCs would likely overcome their detrimental effects.

Second, the expanded cells were relatively incapable of stimulating T cells. Compared with conventional DCs, the expanded cells expressed reduced levels of proinflammatory cytokines (Fig. 2D) and adherence molecules (Fig. 2E). Furthermore, the expanded cells showed almost undetectable levels of MHC molecule I-A and costimulatory molecules (Fig. 2E), which remained stable even after *in vitro* exposure to LPS (Fig. 2F). Consistently, the expanded cells were less potent at inducing IL-17A-producing T cells than conventional DCs *in vitro* (Fig. 3B). While conventional DCs slightly enhanced the antigen-nonspecific proliferation of CD4^+^ T cells *in vitro*, the expanded cells exhibited the opposite effect (Fig. 3D). Thus, the relative incapability of the expanded cells to stimulate T cells should allow them to exert better protection against colitis induced by CD4^+^ CD25^−^ T cells than conventional DCs.

Third, and possibly most importantly, the expanded cells induce T cell apoptosis. More apoptosis was observed in the Payer’s patches (Fig. 7A) and in colonic tissue-infiltrating cells (Fig. 7B) of mice treated with the expanded cells than in those treated with conventional DCs. Induction of T cell apoptosis by the expanded cells was also observed *in vitro* (Fig. 3E; Supplementary Figure 4), and this was reversed by treatment with a neutralisation antibody against FasL (Fig. 4D,E). Although some DC subsets have been reported to induce T cell apoptosis or death, rare studies have reported this ability in regulatory DCs. Splenic CD8a^+^ DCs expressing high levels of FasL can kill CD4^+^ T cells via Fas-mediated apoptosis^20^. Interestingly, the expanded CD11c^low^CD45RB^+^ regulatory DCs showed no CD8a expression (Fig. 2A), and this result is similar to splenic stroma-educated regulatory DCs, which also express FasL and induce apoptosis of activated T cells without expressing CD8a^18^. These findings suggest that induction of T cell apoptosis is one of the mechanisms by which regulatory DCs exert their immunoregulatory function. The induction of T cell apoptosis by the expanded cells is another mechanism that helps them to protect against colitis induced by CD4^+^ CD25^−^ T cells better than conventional DCs.

The mechanism(s) by which endotoxic shock promotes the production of CD11c^low^CD45RB^+^ cells remain elusive. As we failed to detect the *in vitro* expansion of naturally occurring CD11c^low^CD45RB^+^ DCs in the presence of LPS (data not shown), the crosstalk of this cell type with other cell types must be essential for their *in vivo* expansion and for the functional switch after exposure to LPS. Moreover, our data suggest that the intensity of inflammation is indispensable for the expansion of these cells (Fig. 1). Another possible origin of the expanded CD11c^low^CD45RB^+^ cells is *de novo* induction of haematopoietic progenitor cells. Future studies are required to clarify the origin of CD11c^low^CD45RB^+^ cells.

The advantages and disadvantages of these expanded regulatory DCs in various inflammatory diseases are of interest. It is known that the incidences of Th1- and Th17-type inflammatory diseases—type I diabetes mellitus, multiple sclerosis and Crohn’s disease—have increased considerably over the past half century, especially in Europe and North America^25^. It has been suggested that the lack of exposure of persons to sublethal doses of endotoxin due to improved sanitation in industrialised and urban areas increases the incidence of such diseases^25^. It is possible that exposure to sublethal doses of endotoxin leads to the generation or enhancement of certain regulatory mechanism(s) that protect against such diseases. In this regard, it is more important to determine the characteristics of the expanded CD11c^low^CD45RB^+^ cells than those of their naturally occurring counterparts. Our data show the expanded cells can last for a significant amount of time after endotoxic shock (Supplementary Figure 6) and consequently might provide some protection against Th1- and Th17-type inflammatory diseases. In contrast, during sepsis, CD11c^low^CD45RB^+^ cells may not be beneficial because of their extraordinary ability to induce CD4 T cell apoptosis, which could promote immune suppression in the host, making the host more vulnerable to secondary infection^26^.

## Methods

### Mice

C57BL/6, CB-17 SCID, and BALB/c (H-2d) female mice of 8-12 weeks of age were purchased from Beijing Vital River Laboratory Animal, Inc. (Beijing, China, http://www.vitalriver.com.cn). All mice were maintained under specific pathogen-free conditions. The care, use and treatment of mice in this study were in strict agreement with the guidelines set by the Institute of Basic Medical Sciences. Briefly, mice were anaesthetised by administrating pentobarbital i.p. at 50 mg/kg and were subjected to euthanasia by cervical dislocation. All *in vivo* experiments were in accordance with the ARRIVE guidelines, and all efforts were made to minimise the suffering of the mice. The food and water provided to the mice were subjected to aseptic processing. The number of mice used in this study was approximately 150, and all mice were euthanised.

### Induction of peritonitis

Log-phase *E. coli* K12 were washed and resuspended in 0.5 ml of PBS to the desired concentration. Each mouse received an i.p. injection of 1 × 10^8^ cfu *E. coli* K12 or 2 ml of thioglycolate per 20 g of body weight.

### Flow cytometry

Cells from spleens and lymph nodes were depleted of erythrocytes by hypotonic lysis. The cells were washed with FACS washing buffer (2% FBS, 0.1% NaN_3_ in PBS) twice and were then incubated with fluorescence-conjugated antibodies against cell surface molecules for 30 min on ice in the presence of 2.4G2 mAb to block FcγR binding. Isotype antibodies were included as negative controls. For intracellular cytokine staining, single-cell suspensions were stimulated with 50 ng/ml PMA and 1 μM ionomycin in the presence of brefeldin A solution for 4 h. After stimulation, cells were stained with fluorescence-conjugated antibodies against CD4 and CD25, fixed and permeabilised using a fixation/permeabilisation kit (eBioscience, San Diego, CA, USA) and stained with fluorescence-conjugated specific antibodies against IFN-γ, IL-17A, and Foxp3 in accordance with the manufacturer’s instructions. Flow cytometry was performed using a Becton Dickinson FACSCalibur machine.

### Purification of DC subsets

First, DCs were enriched using an OptiPrep density gradient (Axis-Shield PoC AS, Oslo, Norway) by centrifugation at 600 × *g* for 30min at 24 °C. This protocol generated more than 30% CD11c^+^ cells. The mixture was stained with fluorescence-conjugated antibodies against CD11c, CD45RB, and MHC molecule I-A. Different subsets of DCs were then sorted using a Becton Dickinson FACSVantage machine. Cell purity was verified to be at least 95%.

### Giemsa staining

Cells were collected on precoated (poly-L-lysine) coverslips, fixed with methanol, and stained with Giemsa dye for 10 min. After washing with water, the morphology of these cells was observed under a microscope.

### Purification of CD4^+^ CD25^−^ T cells

First, CD4^+^ T cells were isolated by negative selection from single-cell suspensions of spleens using a CD4^+^ T cell isolation kit II (Miltenyi Biotech, Bergisch Gladbach, Germany). Then, the cells were incubated with CD25 microbeads, and all CD25^+^ cells were eliminated using an MS column. The purity of CD4^+^ CD25^−^ T cells was >95% as confirmed by flow cytometry.

### *In vitro* assays for T cell differentiation

Purified CD11c^low^CD45RB^+^ cells or CD11c^hi^CD45RB^−^ cells were co-cultured with CD4^+^ CD25^−^ splenic cells (2.5 × 10^4^ cells/well) in a 96-well plate at a ratio of 4:1. For the Th0 condition, the cells were stimulated with Dynabeads mouse CD3/CD28 T cell expanders (Invitrogen, Carlsbad, CA, USA) in the presence of neutralisation antibodies against IFN-γ and IL-4 (1 μg/ml each) for 5 days. For the Th1 condition and the Treg condition, 10 ng/ml recombinant murine IL-12 and 10 ng/ml recombinant human TGF-β were used in addition to the reagents used for the Th0 condition, respectively. The supernatants were harvested for ELISA. CD4^+^ T cell differentiation of cells was assessed by flow-cytometric analysis of IFN-γ, IL-17A, and Foxp3.

### *In vitro* assays for T cell proliferation and apoptosis

Purified DC subsets were co-cultured with CD4^+^ CD25^−^ splenic cells as stated above. 72 or 96 h later, cells were stained with anti-CD4-PECy5, PI, and Annexin V-FITC resuspended in 300 μl binding buffer containing calcium ion. Apoptosis was assessed by flow-cytometric analysis of Annexin-V/PI staining in CD4^+^ cells. Cells were collected for the same time period and the number of CD4^+^ Annexin-V^−^ cells was counted. For the analysis of T cell proliferation, CD4^+^ CD25^−^ splenic cells at 10^6^/ml were labelled by incubation in RPMI 1640 medium with 0.1 μM CFSE at 37 °C for 20 min before co-culturing them with DCs. Proliferation was assessed by flow-cytometric analysis of CFSE dilutions of CD4^+^ cells after 96 h of co-culture.

### Real-time PCR

RNA was extracted by using TRIzol reagent. First-strand synthesis was performed with Oligo dT primers and reverse transcription was performed with M-MLV reverse transcriptase. Quantitative real-time PCR was performed using SYBR Green reagent (TOYOBO, Tokyo, Japan) in a real-time PCR machine Realplex 2 (Eppendorf, Hamberg, Germany). Reactions were performed three times independently, and GAPDH values were used to normalise gene expression. The following primers were used: murine iNOS, 5′-gttctcagcccaacaatacaaga-3′ (forward) and 5′-gtggacgggtcgatgtcac-3′ (reverse); murine arginase 1, 5′-ctccaagccaaagtccttagag-3′ (forward) and 5′-aggagctgtcattagggacatc-3′ (reverse); and GAPDH, 5′-ggcaaattcaacggcacagt-3′ (forward) and 5′-agatggtgatgggcttccc-3′ (reverse).

### ROS production assays

A LIVE Green Reactive Oxygen Species Detection Kit (Life Technologies, Eugene, OR, USA) was used for detecting the generation of ROS. Briefly, cells were incubated in serum-free RPMI medium containing 2 μM carboxy-H_2_DCFDA, anti-CD11c-PECy5 and anti-CD45RB-PE at 37 °C for 30 min. Cells were washed with PBS and were immediately subjected to flow cytometry to analyse the intensity of green fluorescence at a 488 nm excitation wavelength.

### Induction of colitis by transfer of CD4^+^ CD25^−^ T cells

Mice within litters were randomly divided into different groups. Each litter (containing mice in different groups) was housed in the same cage. Induction of colitis by the transfer of CD4^+^ CD25^−^ T cells was performed according to methods described in a previous report, with some modifications^18^,^19^. Briefly, CD4^+^ CD25^−^ T cells (3 × 10^5^ cells/mouse) obtained from BALB/c mice were suspended in 0.2 ml PBS and i.v. injected into SCID mice. SCID controls received 0.2 ml PBS alone. The day of this transfer was designated as day 0. Then, also on day 0, DCs (1 × 10^6^ cells/mouse) generated from BALB/c mice were injected i.v. The body weights of all mice were measured weekly.

### Histological assessment of colitis induced by transfer of CD4^+^ CD25^−^ T cells

Mice were euthanised, and colons were removed 4 weeks after cell transfer. The transverse colons were fixed for 24 hours in 4% paraformaldehyde, dehydrated, infiltrated with paraffin and sectioned at 5 μm. Slides were stained with haematoxylin/eosin. The grade of inflammation was scored as follows: 1) degree of cell proliferation: 0, none; 1, mild cell number increase and crypt length; 2, moderate cell number increase or focally marked increase; 3, marked increase in entire field of section. 2) severity of inflammation: 0, none; 1, mild lymphocyte infiltration; 2, massive lymphocyte infiltration or visible focal degeneration of crypts; 3, multifocal crypt degeneration and/or tissue structure destruction. 3) extent of inflammation: 0, none; 1, to the mucosal layer; 2, to the submucosal layer; 3, to the transmural layer. 4) amount of mucus: 0, normal; 1, slight decrease of mucus; 2, mild decrease or focal absence of mucus; 3, severe absence of mucus; 4, total absence of mucus. The cumulative histological score was calculated as the sum of the four individual parameters.

### ELISA

The levels of cytokines in the supernatants were determined using ELISA kits according to the manufacturer’s protocols. ELISA kits for IL-6, IL-17A, and TNF-α were from eBioscience (San Diego, CA, USA). ELISA kits for IL-4, IL-10, IFN-γ, and TGF-β were from R&D Systems (Minneapolis, MN, USA).

### Immunohistochemistry

Colons were removed from euthanised mice 4 weeks after cell transfer. The transverse colons were fixed overnight in 4% paraformaldehyde, dehydrated, infiltrated with paraffin and sectioned at 5 μm. Immunohistochemistry was performed using standard protocols with citrate buffer (pH 6.0) pretreatment. Briefly, the sections were incubated with a primary antibody against IFN-γ at 4 °C overnight and then with a corresponding horseradish peroxidase-conjugated secondary antibody at 37 °C for 30 min. Immunohistochemical detection of apoptosis was performed using a TUNEL assay kit (Promega, Madison, WI, USA) according to the manufacturer’s protocol. The sections were finally counterstained with haematoxylin for detection.

### Statistical analysis

The data were shown as mean ± standard deviations (SD). Student’s *t* test was employed to determine significance between two groups (paired or unpaired) and One Way Anova analysis was used to determine significance among several groups. Differences were considered statistically significant when *P* < 0.05.

### Ethics statement

All experimental protocols used in this work were approved by the institutional review board of the Institute of Basic Medical Sciences.

## Additional Information

**How to cite this article**: Wang, X. *et al.* Endotoxic shock-expanded murine CD11c^low^CD45RB^+^ regulatory dendritic cells modulate inflammatory T cell responses through multiple mechanisms. *Sci. Rep.* **5**, 10653; doi: 10.1038/srep10653 (2015).

## Supplementary Material

Supplementary Figures

## References

[b1] Montminy S. W. *et al.* Virulence factors of *Yersinia pestis* are overcome by a strong lipopolysaccharide response. Nat. Immunol. 7, 1066–1073 (2006). 16980981 10.1038/ni1386

[b2] Medzhitov R. Toll-like receptors and innate immunity. Nat. Rev. Immunol. 1, 135–145 (2001). 11905821 10.1038/35100529

[b3] Janeway C. A. Jr & Medzhitov R. Innate immune recognition. Annu. Rev. Immunol. 20, 197–216 (2002). 11861602 10.1146/annurev.immunol.20.083001.084359

[b4] Morelli A. E. & Thomson A. W. Tolerogenic dendritic cells and the quest for transplant tolerance. Nat. Rev. Immunol. 7, 610–621 (2007). 17627284 10.1038/nri2132

[b5] Matsushita H. *et al.* Endotoxin tolerance attenuates airway allergic inflammation in model mice by suppression of the T-cell stimulatory effect of dendritic cells. Int. Immunol. 22, 739–747 (2010). 20584764 10.1093/intimm/dxq062

[b6] Wakkach A. *et al.* Characterization of dendritic cells that induce tolerance and T regulatory 1 cell differentiation *in vivo*. Immunity 18, 605–617 (2003). 12753738 10.1016/s1074-7613(03)00113-4

[b7] Fujita S. *et al.* Regulatory dendritic cells act as regulators of acute lethal systemic inflammatory response. Blood 107, 3656–3664 (2006). 16410444 10.1182/blood-2005-10-4190

[b8] Svensson M., Maroof A., Ato M. & Kaye P. M. Stromal cells direct local differentiation of regulatory dendritic cells. Immunity 21, 805–816 (2004). 15589169 10.1016/j.immuni.2004.10.012

[b9] Liu Q., Yao Y., Zhang S., Yan Y. & Wu X. Naturally existing CD11c^low^CD45RB^high^ dendritic cells protect mice from acute severe inflammatory response induced by thermal injury. Immunobiology 216, 47–53 (2011). 20392518 10.1016/j.imbio.2010.03.005

[b10] Wong K. A., & Rodriguez A. *Plasmodium* infection and endotoxic shock induce the expansion of regulatory dendritic cells. J. Immunol. 180, 716–726 (2008). 18178809 10.4049/jimmunol.180.2.716 PMC2560172

[b11] Keul P. *et al.* Sphingosine-1-phosphate receptor 3 promotes recruitment of monocyte/ macrophages in inflammation and atherosclerosis. Circ. Res. 108, 314–323 (2011). 21164103 10.1161/CIRCRESAHA.110.235028

[b12] Braun M. C., He J., Wu C. Y. & Kelsall B. L. Cholera toxin suppresses interleukin (IL)-12 production and IL-12 receptor β1 and β2 chain expression. J. Exp. Med. 189, 541–552 (1999). 9927516 10.1084/jem.189.3.541 PMC2192916

[b13] Wykes M. N. *et al.* Rodent blood-stage *plasmodium* survive in dendritic cells that infect naïve mice. Proc. Natl. Acad. Sci. USA 108, 11205–11210 (2011). 21690346 10.1073/pnas.1108579108 PMC3131307

[b14] Benoist C. & Mathis D. Autoimmunity provoked by infection: how good is the case for T cell epitope mimicry? Nat. Immunol. 2, 797–801 (2001). 11526389 10.1038/ni0901-797

[b15] Panoutsakopoulou V. & Cantor H. On the relationship between viral infection and autoimmunity. J. Autoimmun. 16, 341–345 (2001). 11334502 10.1006/jaut.2000.0480

[b16] Dalwadi H., Wei B., Kronenberg M., Sutton C. L. & Braun J. The Crohn’s disease-associated bacterial protein I2 is a novel enteric t cell superantigen. Immunity 15, 149–158 (2001). 11485746 10.1016/s1074-7613(01)00164-9

[b17] Marrack P., & Kappler J. Control of T cell viability. Annu. Rev. Immunol. 22, 765–787 (2004). 15032596 10.1146/annurev.immunol.22.012703.104554

[b18] Xu X. *et al.* Splenic stroma-educated regulatory dendritic cells induce apoptosis of activated CD4 T cells via Fas ligand-enhanced IFN-γ and nitric oxide. J. Immunol. 188, 1168–1177 (2012). 22205032 10.4049/jimmunol.1101696

[b19] Rodriguez P. C. *et al.* Arginase I production in the tumor microenvironment by mature myeloid cells inhibits T-cell receptor expression and antigen-specific T-cell responses. Cancer Res. 64, 5839–5849 (2004). 15313928 10.1158/0008-5472.CAN-04-0465

[b20] Suss G. & Shortman K. A subclass of dendritic cells kills CD4 T cells via Fas/Fas-ligand-induced apoptosis. J. Exp. Med. 183, 1789–1796 (1996). 8666935 10.1084/jem.183.4.1789 PMC2192509

[b21] Kjellev S., Lundsgaard D., Poulsen S. S. & Markholst H. Reconstitution of Scid mice with CD4 + CD25- T cells leads to rapid colitis: an improved model for pharmacologic testing. Int. Immunopharmacol. 6, 1341–1354 (2006). 16782548 10.1016/j.intimp.2006.04.017

[b22] Yamanishi H. *et al.* Regulatory dendritic cells pulsed with carbonic anhydrase I protect mice from colitis induced by CD4 + CD25- T cells. J. Immunol. 188, 2164–2172 (2012). 22291189 10.4049/jimmunol.1100559

[b23] Elinav E., Waks T. & Eshhar Z. Redirection of regulatory T cells with predetermined specificity for the treatment of experimental colitis in mice. Gastroenterology 134, 2014–2024 (2008). 18424268 10.1053/j.gastro.2008.02.060

[b24] Yamazaki S. *et al.* CD8 + CD205 + splenic dendritic cells are specialized to induce Foxp3 + regulatory T cells. J. Immunol. 181, 6923–6933 (2008). 18981112 10.4049/jimmunol.181.10.6923 PMC2814590

[b25] Bach J. F. The effect of infections on susceptibility to autoimmune and allergic diseases. N. Engl. J. Med. 347, 911–920 (2002). 12239261 10.1056/NEJMra020100

[b26] Gurung P. *et al.* Immune unresponsiveness to secondary heterologous bacterial infection after induction is TRAIL dependent. J. Immunol. 187, 2148–2154 (2011). 21788440 10.4049/jimmunol.1101180 PMC3159846

