# Supplementary material for: Endotoxic shock-expanded murine CD11clowCD45RB+ regulatory dendritic cells modulate inflammatory T cell responses through multiple mechanisms
Source: Sci Rep. 2015 May 29;5:10653. doi: 10.1038/srep10653 (PMC4448501; doi:10.1038/srep10653)
Supplement: Supplementary Figures [file srep10653-s1.doc]

**Supplementary information**

**Endotoxic shock-expanded murine CD11c^low^CD45RB^+^ regulatory dendritic cells modulate inflammatory T cell responses through multiple mechanisms**

Qingyang Wang^1^, Xueying Zhang^1^, Xiaoqian Wang^1,2^, Yajing Li^1,2^, Jingyang Wang^1^, Chunmei Hou^1^, Juan Chen^1^, Beifen Shen^1^, Yanchun Shi^2^ and Jiyan Zhang^1,*^

**Supplementary Figures**

**Supplementary Figure 1**. The expression of MHC molecule I-A on the surface of CD11c^low^CD45RB^+^ cells in untreated mice was analysed by flow cytometry. The red line represents cells stained with the isotype antibody.

**Supplementary Figure 2**. CD11c^low^CD45RB^+^I-A^-^ cells in untreated mice were purified. The expression of MHC molecule I-A before (red) and after (blue) stimulation with 0.5 μg/ml LPS for 24 h was analysed by flow cytometry.

**Supplementary Figure 3**. Purified CD11c^low^CD45RB^+^ cells or CD11c^hi^CD45RB^-^ cells were co-cultured with CD4^+^CD25^-^ splenic cells at the ratio of 4:1. Stimulation was affected by Dynabeads mouse CD3/CD28 T cell expander. The co-culture was subjected to Th0 condition. 5 days later, Foxp3 expression in CD4^+^ cells was analyzed by flow cytometry.

**Supplementary Figure 4**. Purified CD11c^low^CD45RB^+^ cells and CD11c^hi^CD45RB^-^ cells were co-cultured with CD4^+^CD25^-^ splenic cells at the ratio of 4:1. Stimulation was affected by Dynabeads mouse CD3/CD28 T cell expander. 72 h later, cells were stained with anti-CD4-PECy5, PI, and Annexin V-FITC resuspended in 300 μl binding buffer containing calcium ion. Apoptosis was assessed by flow-cytometric analysis of the percentages of CD4^+^Annexin-V^+^ cells.

**Supplementary Figure 5**. DCs (1 × 10^6^ cells/mouse) generated from BALB/c mice or PBS of equal volume were injected i.v. into SCID mice. Four weeks later, the splenic cells were subjected to flow cytometry analysis of CD4 or CD3 expression.

**Supplementary Figure 6**. BALB/c mice were i.p. injected with *E. coli*-derived LPS. After different periods of time, the splenic cells or MLN cells were subjected to flow cytometry analysis of CD11c and CD45RB expression. The percentages of CD11c^int^CD45RB^+^ cells were shown.
